# Nucleus Accumbens and Ventral Tegmental Area Functional Connectivity as a Marker of Reward Sensitivity in Infancy

**DOI:** 10.1111/desc.70277

**Published:** 2026-09-03

**Authors:** Megan M. Hare, Yanbin Niu, Charles H. Zeanah, Kathryn L. Humphreys

**Affiliations:** ^1^ Department of Psychology and Human Development, Peabody College Vanderbilt University Nashville Tennessee USA; ^2^ Department of Psychiatry and Behavioral Sciences Tulane University School of Medicine New Orleans Louisiana USA

**Keywords:** infant MRI, nucleus accumbens, reward circuitry, reward responsiveness, ventral tegmental area

## Abstract

Early functional connectivity within the brain's reward circuitry may provide insight into the developmental origins of individual differences in pleasure and motivation. This study examined resting‐state functional connectivity between two key reward‐related regions, the nucleus accumbens (NAc) and ventral tegmental area (VTA), in 94 one‐month‐old infants (58% male; M = 4.84 weeks, *SD* = 0.86) and its association with later reward‐related temperament. Infants completed a resting‐state functional magnetic resonance imaging scan during natural sleep with a mean of 11.09 (*SD* = 2.78) min of retained low‐motion data. Caregivers reported their infants’ temperament at age six months, including high‐ and low‐intensity pleasure as indicators of individual differences relevant to reward sensitivity. Positive resting‐state functional connectivity between the NAc and VTA was present in early infancy (Cohen's *d* = 0.44, 95% CI [0.22, 0.65]), p < 0.001). Less positive NAc–VTA connectivity was associated with greater high‐intensity pleasure at six months (β = ‐0.32, 95% CI [‐0.54, ‐0.10], *p* = 0.004), indicating that infants with stronger connectivity between these regions exhibited less enjoyment of highly stimulating, energetic activities. NAc–VTA connectivity was not associated with low‐intensity pleasure (β = ‐0.01, 95% CI [‐0.24, 0.21], *p* = 0.908). These findings suggest that early variation in mesolimbic connectivity may serve as a marker of emerging individual differences in reward responsiveness, with implications for understanding affective development in infancy.

## Summary

- Examines resting‐state functional connectivity between the nucleus accumbens and ventral tegmental area in one‐month‐old infants.
- Identifies early evidence of positive mesolimbic connectivity in the first weeks after birth.
- Links more positive NAc–VTA connectivity to lower high‐intensity pleasure at six months.
- Highlights early individual differences in reward circuitry as potential markers of affective development.

## 1 Introduction

A recent developmental model conceptualizes reward processing as comprising separable components, including association, discrimination, preference and valuation, effort, anticipation, and response, that emerge on different timetables across infancy and childhood (Clements et al. 2023). From the first months of life, infants display individual differences in these behaviors, including approach behavior, positive affect, and engagement with pleasurable stimuli, which shape how they interact with caregivers and their environment (Gartstein and Rothbart 2003; Planalp et al. 2017). These early expressions of pleasure are considered core components of temperament and reflect variation in affective reactivity and motivational tendencies during a period of rapid neurodevelopment (Gartstein and Rothbart 2003; Rothbart et al. 2000). Accordingly, linking early behavioral expressions of pleasure to the neural systems that support emerging motivational processes provides a developmentally grounded rationale for examining the functional organization of mesolimbic circuitry in infancy.

### 1.1 Developmental Organization of Reward Circuitry in Early Life

The mesolimbic dopamine system, particularly the ventral tegmental area (VTA) and nucleus accumbens (NAc), plays a central role in motivation and reward‐related behavior across species (Haber and Knutson 2010; Russo and Nestler 2013; Salamone and Correa 2012). The VTA provides dense dopaminergic projections to the NAc, forming a core circuit involved in reinforcement, approach behavior, and hedonic responding (Berridge and Kringelbach 2015; Ikemoto 2007; O'Doherty 2004). Evidence from developmental neuroscience indicates that core components of the mesolimbic system are present early in life. Anatomical studies demonstrate that dopaminergic projections from the VTA to the striatum, including the NAc, are established prenatally and undergo rapid maturation during the early postnatal period (Hou et al. 2024; Voorn et al. 1988).

Non‐human animal research provides specific evidence that developing dopamine circuits contribute to social approach early in life. In infant rats, experimental activation and inhibition of dopaminergic projections from the VTA to the basolateral amygdala bidirectionally altered social approach toward the caregiver, demonstrating a causal role for this developing pathway in infant social behavior (Opendak et al. 2021). These findings indicate that dopamine circuitry is behaviorally functional during infancy, although they do not establish that the canonical VTA–NAc pathway already performs the mature reward‐learning functions observed later in development (Opendak et al. 2025). Human infant neuroimaging studies provide converging support for early functional organization of subcortical systems. Resting‐state fMRI research demonstrates that newborns and young infants exhibit coherent functional connectivity within subcortical and cortico‐subcortical networks implicated in salience, reward, and socioemotional processing (De Asis‐Cruz et al. 2015; Fransson et al., 2009; Gao et al. 2017). A resting‐state network encompassing the bilateral basal ganglia has been identified in full‐term newborns, indicating that coordinated spontaneous activity involving striatal regions is detectable shortly after birth (Fransson et al., 2009). Despite these advances, connectivity between the NAc and VTA, one of the core pathways of the mesolimbic system, remains largely understudied in infancy.

### 1.2 NAc–VTA Connectivity Across Development

Across species and developmental stages, functional coupling between the NAc and VTA is central to motivational and affective processes. Preclinical studies demonstrate reciprocal regulation within the VTA–NAc circuit: VTA GABAergic projections to the NAc influence reinforcement, whereas GABAergic projections from the NAc back to the VTA modulate dopamine activity and cocaine‐related reward behavior (Al‐Hasani et al. 2021; Khayat and Yaka 2024). In humans, functional connectivity between these regions is reliably observed during both reward‐related tasks and resting‐state paradigms, indicating that NAc–VTA coupling represents a core feature of the brain's motivational circuitry (Haber and Knutson 2010; Murty et al. 2018). The functional significance of this circuitry likely differs across developmental stages.

In adults and adolescents, VTA–NAc function is frequently studied in relation to reward anticipation, incentive motivation, and reinforcement learning. In infancy, however, the behavioral function of VTA–NAc connectivity remains unknown, and adult‐defined reward functions should not be assumed to apply to the immature circuit (Clements et al. 2023; Opendak et al. 2025). Importantly, prior work in older children suggests that associations between mesolimbic connectivity and reward‐related behavior vary across development. For example, adolescents often show heightened mesolimbic engagement during reward processing relative to adults, consistent with developmental models of increased reward sensitivity during youth (Galván 2010; van Duijvenvoorde et al. 2016). Such variability may reflect differences in developmental stage, sample characteristics, reward type, and methodological approach; early in development, mesolimbic circuitry may primarily support affective engagement and arousal modulation, whereas later these circuits become increasingly specialized for reinforcement learning and goal‐directed reward seeking. The developmental origins of these patterns remain unclear, as very few studies have examined this circuitry during infancy. Studying NAc–VTA connectivity early in life is therefore informative for determining whether individual differences in mesolimbic organization emerge shortly after birth and whether they are already linked to observable variation in affective behavior.

### 1.3 Mapping Reward Circuitry to Temperament‐Based Expressions of Pleasure

In infancy, reward‐related tendencies are assessed through temperament measures capturing expressions of positive affect and enjoyment in response to environmental stimulation (Gartstein and Rothbart 2003; Putnam et al. 2014). Two relevant dimensions are high‐intensity pleasure, reflecting enjoyment of stimulating, energetic activities (e.g., active play), and low‐intensity pleasure, reflecting enjoyment of calm, low‐arousal experiences (e.g., quiet play). Prior longitudinal research indicates that positive affectivity and surgency‐related traits, including high‐intensity pleasure, show moderate stability across infancy and early childhood (Putnam et al. 2008). In contrast, comparatively less research has examined the developmental stability of low‐intensity pleasure specifically, and existing evidence is more limited, suggesting these dimensions may follow partially distinct developmental trajectories. Although both domains index positive affect, they differ in arousal context and behavioral expression, suggesting potentially distinct underlying neurobiological mechanisms. Because these temperament scales capture emotional expressiveness across different levels of stimulation, examining their associations with NAc–VTA connectivity may clarify how early mesolimbic organization contributes not only to reward‐related responding, but also to the modulation of positive affect in infancy. Prior research indicates that mesolimbic regions, particularly the NAc, are especially responsive to salient and high‐arousal rewards, supporting the hypothesis that neural variability within this circuitry may be more strongly associated with behaviors reflecting high‐arousal engagement than with calmer forms of positive affect.

Conceptually, high‐intensity pleasure may be more closely aligned with approach‐related motivation and behavioral exuberance in response to salient or stimulating rewards, processes linked to mesolimbic dopamine function and striatal sensitivity to high‐arousal incentives (Bjork et al. 2004; Sescousse et al. 2013). In contrast, low‐intensity pleasure reflects positive affect in low‐arousal contexts and may rely more heavily on broader socioemotional and regulatory processes (Gartstein et al. 2017; Rothbart 2011). Because the NAc is particularly sensitive to salient and high‐arousal rewards, associations with high‐intensity pleasure would be expected to be stronger than those with low‐intensity pleasure, making it informative to examine these dimensions separately. Importantly, in early infancy, reward‐related functioning is expressed primarily through observable affective engagement and enjoyment of stimuli rather than through mature reinforcement‐learning mechanisms. Accordingly, linking mesolimbic circuitry to temperament‐based expressions of positive affect provides a developmentally grounded approach to studying early motivational systems.

### 1.4 The Current Study

The present study aimed to advance understanding of the early neural foundations of reward‐related affect by examining resting‐state functional connectivity between the NAc and VTA in one‐month‐old infants and its prospective association with caregiver‐reported expressions of pleasure at six months. First, we characterized the presence and magnitude of NAc–VTA connectivity in early infancy, a developmental period during which subcortical circuits are functionally active but still undergoing rapid maturation. Second, we tested whether individual differences in mesolimbic connectivity were associated with high‐ and low‐intensity pleasure, conceptualized as temperament‐based expressions of positive affect in high‐ versus low‐arousal contexts. Given prior mixed findings regarding the direction of associations between mesolimbic connectivity and reward‐related behavior across developmental stages, and the limited infant literature, we did not make a strong directional hypothesis regarding whether NAc–VTA connectivity would be positively or negatively associated with pleasure. However, based on evidence that mesolimbic circuitry is particularly responsive to salient and high‐arousal rewards, we anticipated that any associations with early connectivity would be more evident for high‐intensity pleasure than for low‐intensity pleasure.

## 2 Methods

### 2.1 Participants and Procedures

Families were recruited during pregnancy from the greater the greater Nashville, Tennesseearea through obstetrics clinics and social media advertisements for a larger longitudinal study of infant brain and behavioral development. Interested individuals were first screened via phone to confirm eligibility, which included being 18 years or older, currently pregnant with a singleton pregnancy, fluent in English, a U.S. citizen or permanent resident, and having no immediate plans to move out of the greater the greater Nashville, Tennessee area given the longitudinal nature of the study. After their due date, participants were screened again to assess their infant's eligibility for MRI scanning. Exclusion criteria included caregiver‐reported severe complications during birth, infant head trauma, premature birth (prior to 36 weeks gestation), and any infant MRI contraindications (e.g., metal implants). All study procedures were approved by the Vanderbilt University Institutional Review Board. Informed consent was obtained from all participants, including consent for their infant's participation following birth. Eligible infants underwent MRI scanning during natural sleep when they were approximately one month old while mothers completed a battery of questionnaires. When infants were approximately six months old, mother–infant dyads completed a laboratory visit in which they participated in mother–infant interactions and mothers completed questionnaires. Questionnaire data were collected and managed using REDCap electronic data capture tools hosted at Vanderbilt University (Harris et al. 2009, Harris et al. 2019).

Of the 328 participants who enrolled in the study during pregnancy, MRI scanning was attempted with 142 infants at one month of age. Structural MRI and rsfMRI scans were successfully acquired from 111 infants. Of these infants, 94 provided high‐quality rsfMRI data (i.e., scans with at least five minutes of low motion data), comprising the final analyzable sample for the current study. After censoring, the mean framewise displacement (FD) was 0.12 mm (*SD* = 0.03), and an average of 11.09 min (SD = 2.78) of low‐motion data were retained. There were no significant demographic differences between participants included in the analyses and those excluded. See Table 1 for sample characteristics.

**TABLE 1:** Sample demographic characteristics.

Infant sex (%)	58% Males
	42% Females
Infant age at scan (Mean, SD, range)	4.84 weeks (*SD* = 0.86; range: 3.43–6.86 weeks)
Infant race (%)	
American Indian or Alaska Native	0%
Asian	2%
Black or African American	6%
Native Hawaiian or Other Pacific Islander	0%
White	82%
Other	5%
Infant ethnicity (%)	7% Hispanic/Latinx
Caregiver education (%)	
High school graduate or equivalent	2%
Trade/technical/vocational training	2%
Some college credit, no degree	7%
Associate's degree	2%
Bachelor's degree	27%
Graduate degree	57%
Other	2%
Income‐to‐needs ratio (Mean, SD, range)	1.6 (*SD* = 0.76; range: 0.13–2.53)

### 2.2 MRI Acquisition

MR images were acquired at infant age between three and seven weeks at the Vanderbilt Institute of Imaging Science Center for Human Imaging using a Philips Ingenia Elition 3.0 T equipped with a 32‐channel head coil. Infants were prepared using the “swaddle and soothe” method and scanned during natural sleep in the evenings (for more details, see Camacho et al. 2020).

The scans collected included a T2‐weighted anatomical image (TR = 2500 ms, TE = 310 ms, flip angle = 90 degrees, FOV = 220×220×144 mm, resolution = 0.8×0.8×0.8 mm^3^) and resting‐state fMRI (resolution = 2.0×1.9×1.9 mm^3^, 54 axial slices, 96×96 acquisition matrix, flip angle = 60 degrees, TR = 1410 ms, TE = 30 ms, 3 simultaneous slices, 270 contiguous volumes). Framewise Integrated Real‐time MRI monitoring (FIRMM) software was installed by VUIIS partway through data collection and was used to monitor motion in real time for the resting‐state scan and the sequence was repeated until approximately 10‐min of fMRI data under 0.2 mm framewise displacement was collected or until the scan was ended. All MR images were visually inspected for artifacts prior to processing.

### 2.3 MRI Processing

Anatomical segmentation was generated from T2w images with BIBSNet v3.4.1 (Hendrickson et al. 2025) and supplied to NiBabies v24.1.0, derived from fMRIPrep (Esteban et al. 2019), as a precomputed segmentation. The underlying workflow engine used was Nipype 1.8.6 (Esteban et al. 2022; Gorgolewski et al. 2011).

#### 2.3.1 Anatomical Data Preprocessing

The T2w image was denoised and corrected for intensity non‐uniformity (INU). A pre‐computed T2w brain mask was provided as input and used throughout the workflow. Brain surfaces were reconstructed with a modified MCRIBReconAll (M‐CRIB‐S; Adamson et al. 2020) workflow, using the reference T2w and the pre‐computed anatomical segmentation. Volume‐based spatial normalization to two standard spaces (MNIInfant:cohort‐1, MNI152NLin6Asym) was performed through nonlinear registration with antsRegistration (ANTs), using brain‐extracted T2w images. The following templates were selected for spatial normalization and accessed with TemplateFlow (24.2.2; Ciric et al. 2022): MNI Unbiased nonlinear Infant Atlases 0–4.5 years (Fonov et al. 2009), FSL's MNI ICBM 152 non‐linear 6th Generation Asymmetric Average Brain Stereotaxic Registration Model (Evans et al. 2012).

#### 2.3.2 Functional Data Preprocessing

A B0‐nonuniformity map (or fieldmap) was estimated based on echo‐planar imaging (EPI) references with topup (Andersson et al. 2003). For each of the BOLD runs, the following preprocessing was performed. First, a reference volume was generated, using a custom methodology of NiBabies, for use in head motion correction. Head‐motion parameters with respect to the BOLD reference (transformation matrices, and six corresponding rotation and translation parameters) were estimated before any spatiotemporal filtering using mcflirt (FSL, Jenkinson et al. 2002). The estimated fieldmap was then aligned with rigid‐registration to the target EPI (echo‐planar imaging) reference run. The field coefficients were mapped on to the reference EPI using the transform. The BOLD reference was then co‐registered to the anatomical reference using bbregister (FreeSurfer) which implements boundary‐based registration (Greve and Fischl 2009). Co‐registration was configured with six degrees of freedom. The aligned T2w image was used for initial co‐registration. Several confounding time‐series were calculated based on the preprocessed BOLD: framewise displacement (FD), DVARS and three region‐wise global signals. FD was computed using two formulations following Power (absolute sum of relative motions, Power et al. 2014) and Jenkinson (relative root mean square displacement between affines, Jenkinson et al. 2002). FD and DVARS are calculated for each functional run, both using their implementations in Nipype (following the definitions by Power et al. 2014). The three global signals are extracted within the CSF, the WM, and the whole‐brain masks. The confound time series derived from head motion estimates and global signals were expanded with the inclusion of temporal derivatives and quadratic terms for each (Satterthwaite et al. 2013). Frames that exceeded a threshold of 0.5 mm FD or 1.5 standardized DVARS were annotated as motion outliers.

The eXtensible Connectivity Pipeline‐DCAN (XCP‐D; Ciric et al. 2018; Mehta et al. 2023; Satterthwaite et al. 2013) was used to post‐process the outputs of nibabies. XCP‐D was built with Nipype version 1.8.6 (Esteban et al. 2022; Gorgolewski et al. 2011). Native‐space T2w images were transformed to MNIInfant space at 1 mm^3^ resolution. For each of the BOLD runs, the following post‐processing was performed. The six translation and rotation head motion traces were band‐stop filtered to remove signals between 12.55 and 17.45 breaths‐per‐minute (automatically modified from 30.0 and 60.0 BPM due to Nyquist frequency constraints) using a(n) first‐order notch filter, based on (Fair et al. 2020). The Volterra expansion of these filtered motion parameters was then calculated. Framewise displacement was calculated from the filtered motion parameters using the formula from Power et al. (2014), with an automatically estimated head radius. Volumes with filtered framewise displacement greater than 0.2 mm were flagged as high‐motion outliers for the sake of later censoring (Power et al. 2014). In total, 36 nuisance regressors were selected from the preprocessing confounds, according to the ‘36P’ strategy. These nuisance regressors included six motion parameters, mean global signal, mean white matter signal, mean cerebrospinal fluid signal with their temporal derivatives, and quadratic expansion of six motion parameters, tissue signals and their temporal derivatives (Ciric et al. 2018; Satterthwaite et al. 2013). Any motion parameters in the confounds file were filtered using the same parameters as described above and the Volterra expansion was calculated. The BOLD data were despiked with AFNI's 3dDespike. Nuisance regressors were regressed from the BOLD data using a denoising method based on Nilearn's approach. Any volumes censored earlier in the workflow were first cubic spline interpolated in the BOLD data. Outlier volumes at the beginning or end of the time series were replaced with the closest low‐motion volume's values, as cubic spline interpolation can produce extreme extrapolations. The timeseries were band‐pass filtered using a(n) second‐order Butterworth filter, in order to retain signals between 0.01‐0.1 Hz. The same filter was applied to the confounds. The resulting time series were then denoised via linear regression, in which the low‐motion volumes from the BOLD time series and confounds were used to calculate parameter estimates, and then the interpolated time series were denoised using the low‐motion parameter estimates. The interpolated time series were then censored using the temporal mask.

#### 2.3.3 Resting‐State fMRI Network Analysis

**FIGURE 1:**
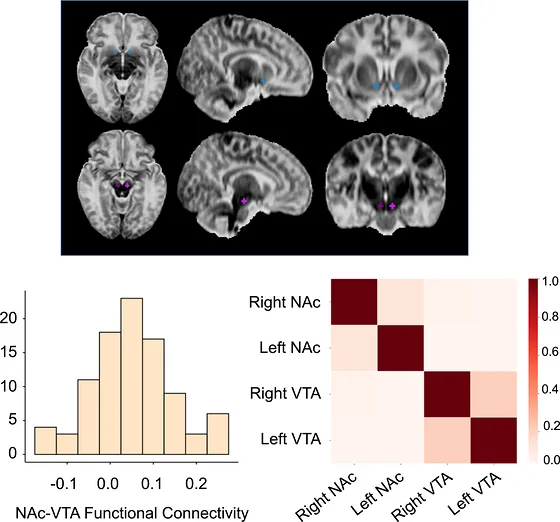
ROI positions and functional connectivity analysis between Nucleus Accumbens (NAc) and Ventral Tegmental Area (VTA). Upper Panel: Regions of interest (ROI) positions for the Nucleus Accumbens (NAc) and Ventral Tegmental Area (VTA). Lower Left: Histogram of NAc–VTA functional connectivity. Lower Right: Mean functional connectivity (Pearson's *r*) between the NAc and VTA.

Regions of interest (ROIs) were created based on the anatomical landmarks visible on the neonate template brain. Spheres (6 mm diameter) were centered on the NAc and the VTA separately for each hemisphere. Specific coordinates for the sphere centers are listed in template space in Table 2 and visualized in Figure 1. Pearson's correlations were computed between each seed for each participant and then normalized using Fisher's Z‐transformation.

**TABLE 2:** Coordinates for the resting state functional MRI connectivity analyses.

	Extent	Coordinates		
Region	mm^3^	X	Y	Z
Left Nucleus Accumbens	56	47	76	33
Right Nucleus Accumbens	56	56	76	33
Left Ventral Tegmental Area	56	48	65	31
Right Ventral Tegmental Area	56	54	65	31

*Note*: Extent refers to the volume of the mask, measured in mm^3^. Coordinates are the spatial location of the center of the mask in template space, represented by the X, Y, and Z coordinates.

#### 2.3.4 Reward Responsiveness at Six Months

Infant reward‐related affect was assessed using the Infant Behavior Questionnaire‐Short Form Revised (IBQ‐R SF) (Putnam et al. 2014), a widely used measure of temperament. Caregivers reported on their infant's behavior over the last 7 days using a 7‐point Likert‐type scale ranging from 1 (*never*) to 7 (*always*), with an additional response option, “Does not apply” for situations not encountered during the past week. The IBQ‐R SF consists of 91 items categorized into 14 scales, two of which were used in the current study: high‐intensity pleasure and low‐intensity pleasure. The high‐intensity pleasure scale measures an infant's enjoyment of high intensity, energetic activities across 7 items (e.g., “How often did the baby enjoy bouncing up and down while on your lap?” and “During a peekaboo game, how often did the baby laugh?”). The low‐intensity pleasure scale measures an infant's pleasure in low, calm activities also across 6 items (e.g., “How often did your baby enjoy listening to a musical toy in a crib?” and “When playing quietly with one of their favorite toys, how often did the baby enjoy lying in the crib for more than 5 min?”). Within our sample, high‐intensity pleasure demonstrated acceptable reliability (α = 0.74), whereas low‐intensity pleasure demonstrated reliability slightly below the conventional cutoff (α = 0.66).

The high‐ and low‐intensity pleasure scales assess infants’ behavioral expressions of enjoyment and positive affect in response to stimulating and low‐arousal contexts, respectively, rather than reward responsiveness in a reinforcement‐learning sense (Gartstein and Rothbart 2003; Putnam et al. 2014). In early infancy, reward‐related processes are expressed primarily through observable pleasure and approach behavior during interactions with the environment, making temperament‐based expressions of enjoyment developmentally appropriate behavioral indicators of early motivational functioning (Clements et al. 2023). Consistent with prior research, the IBQ has demonstrated strong reliability and validity across diverse infant samples, supporting its use as a robust measure of early temperament dimensions (Parade and Leerkes 2008; Peterson et al. 2017; Putnam et al. 2014). Accordingly, we interpret associations between NAc–VTA connectivity and IBQ pleasure as reflecting variation in early reward‐related affective engagement, rather than mature reward processing mechanisms. Importantly, elevated high‐intensity pleasure has been linked to later externalizing behaviors and impulsivity, whereas low‐intensity pleasure is more often interpreted as reflecting contentment and regulatory aspects of positive affect, suggesting that these dimensions may represent distinct facets of early temperament with different developmental implications (Gartstein et al. 2017; Rothbart 2011).

#### 2.3.5 Child Positive Mood

Child positive mood was coded using the Parent–Child Interaction Rating Scales–Infant Adaptation (PCIRS‐IA) (Bosquet Enlow et al. 2014; Sosinsky et al. 2004), a standardized observational coding system that captures qualitative features of parent–infant interaction. Mother–infant dyads completed a 10‐min videorecorded free‐play interaction in the laboratory or via Zoom and were instructed to play as they typically would at home. Trained independent coders rated the infant's general positive affective tone during each 2‐min epoch of the interaction using a 7‐point scale, with higher scores indicating greater positive mood. Twenty‐eight percent of interactions were double‐coded, and interrater reliability was good (ICC = 0.83).

### 2.4 Analytic Plan

All analyses were conducted using *R* version 4.4.0 (R Core Team 2024). All variables were z‐scored prior to analyses to standardize scales across predictors, facilitating interpretation of effect sizes. We used a Pearson's correlation test to examine the association between high and low‐intensity pleasure at age six months. To examine whether individual differences in the magnitude of NAc–VTA connectivity at age one month are associated with later temperamental reward sensitivity, the following linear modeling was implemented through the “lavaan” package (Rosseel 2012). Specifically, we regressed 6‐month high‐intensity pleasure and low‐intensity pleasure, in two separate models, onto one month NAc–VTA resting‐state functional connectivity, using robust maximum likelihood (Huber 1967; White 1982). Average motion (i.e., FD), the duration of low motion resting‐state data, infant age at scanning (corrected for due date), and infant sex were included as covariates. To evaluate the specificity of associations between NAc–VTA connectivity and high and low‐intensity pleasure, we conducted an additional regression model using negative affect as an outcome, reflecting a broader, different temperament dimension. As an exploratory multi‐method analysis, we also examined whether NAc–VTA connectivity was associated with experimenter‐coded child positive mood during the six‐month parent–child interaction.

Of the 94 infants with NAc–VTA connectivity data, 16 participants were missing temperament data. The analyses used full information maximum likelihood (FIML) to address missing data, allowing all available observations, including those with partial outcome data, to contribute to parameter estimation. Missingness was confined to the six‐month outcomes, and estimation assumes data are missing at random conditional on the covariates in the model. Although models were estimated using the structural equation modeling framework in lavaan to enable FIML estimation, each model consisted of a single‐path regression and is mathematically equivalent to a standard multiple linear regression model with the same predictors and covariates. As a robustness check, we also conducted analyses using standard multiple regression restricted to complete cases (n = 78). Results were unchanged in direction and magnitude (see Table S1).

We conducted Grubbs’ test to evaluate whether the most extreme observations deviated from the sample mean beyond what would be expected given the sample size and variance. Two observations for the NAc–VTA connectivity were identified as outliers, with values 2.94 and 4.64 SD from the sample mean. To reduce sensitivity of the analyses to distributional extremes while retaining all observations, values were winsorized to the 5th and 95th percentile thresholds using the winsorize function from the robustHD package (Alfons 2021) in R. Sensitivity analyses conducted without winsorization produced results that were consistent in direction and magnitude, suggesting that these extreme values did not substantively influence the findings (see Table S2).

The sample size was determined by the number of participants in the larger longitudinal study who provided usable resting‐state data, which is typical for neuroimaging studies embedded within larger cohort designs. A sensitivity analysis indicated that with N = 94 and four covariates, the design provided 80% power (α = 0.05) to detect a small‐to‐medium incremental effect of NAc–VTA connectivity (*f* ^2^ ≈ 0.09; partial *r* ≈ 0.29).

## 3 Results

### 3.1 Resting‐State Functional Connectivity Between the NAc and VTA at One Month

At the group level, infants demonstrated statistically significant positive resting‐state functional connectivity between the NAc and VTA (mean Fisher *z* = 0.05, *SD* = 0.11). Connectivity was significantly greater than zero, t(93) = 4.23, *p* < 0.001, Cohen's *d* = 0.44, 95% CI [0.22, 0.65], indicating measurable coupling within the mesolimbic reward pathway by one month of age.

### 3.2 Correlation Between High‐ and Low‐Intensity Pleasure at Six Months

At six months, infants’ mean high‐intensity pleasure score was 5.83 (*SD* = 0.73, range = 3.83–7.00), and their mean low‐intensity pleasure score was 5.57 (*SD* = 0.81, range = 3.00–6.86). The two scales were modestly positively correlated (*r* = 0.27, 95% CI [0.05, 0.47], *p* = 0.015), indicating partial overlap but sufficient distinction to examine them as separate outcomes. Although modestly correlated, these dimensions are theoretically distinct and may index different facets of early affective engagement with rewarding stimuli.

### 3.3 Resting‐State Functional Connectivity Between the NAc and VTA at One Month and Reward Responsiveness at Six Months

**FIGURE 2:**
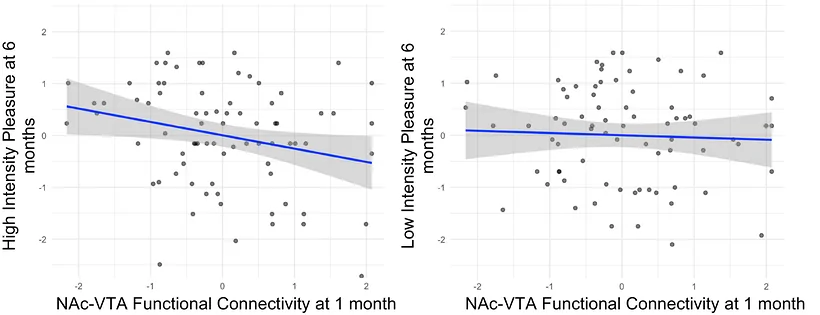
Scatter plot of high‐ and low‐intensity pleasure and functional connectivity between the Nucleus Accumbens (NAc) and Ventral Tegmental Area (VTA). Covariates include average motion (i.e., FD), the length of low motion resting‐state data, infant age at scanning (corrected for due date), and infant sex. Regression lines represent linear associations between standardized variables.

Individual differences in NAc–VTA connectivity at age one month were associated with later variation in reward responsiveness. Specifically, weaker connectivity was associated with greater high‐intensity pleasure at age six months (β = ‐0.32, 95% CI [‐0.54, ‐0.10], *p* = 0.004; see Figure 2), such that infants with more positive connectivity exhibited lower enjoyment of highly stimulating activities. In contrast, connectivity was unrelated to low‐intensity pleasure (β = ‐0.01, 95% CI [‐0.24, 0.21], *p* = 0.908; see Table 3). Together, these findings suggest that NAc–VTA connectivity is detectable in early infancy and prospectively linked to the expression of high‐arousal, but not low‐arousal, positive affect.

**TABLE 3:** Regression results for functional connectivity between the Nucleus Accumbens (NAc) and Ventral Tegmental Area (VTA), and high‐intensity and low‐intensity pleasure.

Model	β	95% CI	SE	z	*p*
Dependent Variable: High‐intensity pleasure at 6‐month					
NAc–VTA FC	−0.32	−0.54, −0.10	0.11	−2.87	0.004
fMRI Motion	0.13	−0.05, 0.30	0.09	1.39	0.166
fMRI Length of Low Motion Data	−0.18	−0.40, 0.05	0.12	−1.52	0.130
Infant Age	0.21	0.01, 0.42	0.10	2.06	0.040
Infant Sex	0.11	−0.09, 0.32	0.10	1.12	0.264

Dependent Variable: Low‐intensity pleasure at 6‐month					
NAc–VTA FC	−0.01	−0.24, 0.21	0.11	−0.12	0.908
fMRI Motion	0.07	−0.15, 0.30	0.12	0.62	0.533
fMRI Length of Low Motion Data	−0.01	−0.23, 0.22	0.12	−0.06	0.954
Infant Age	−0.19	−0.40, 0.02	0.11	−1.78	0.075
Infant Sex	−0.08	−0.29, 0.13	0.21	−0.76	0.446

*Note*: β = fully standardized regression coefficients. *SE*s, *z* statistics, *p*‐values, and 95% CIs were obtained from the fully standardized solution. All continuous variables were z‐scored before analysis. Infant age was assessed at the time of MRI scanning. Infant sex was coded as female = 0 and male = 1 and was not z‐scored before model estimation; its reported coefficient is the fully standardized coefficient.

Abbreviations: FC, functional connectivity; NAc, nucleus accumbens; VTA, ventral tegmental area.

### 3.4 Resting‐State Functional Connectivity Between the NAc and VTA at One Month and Negative Affect at Six Months

To examine the specificity of associations with pleasure‐related behaviors, we conducted additional analyses using negative affect as an outcome. NAc–VTA connectivity was not significantly associated with negative affect (β = −0.23, 95% CI [−0.59, 0.10], *p* = 0.170; Table S3).

### 3.5 Resting‐State Functional Connectivity Between the NAc and VTA at One Month and Experimenter‐Coded Child Positive Mood at Six Months

In an exploratory analysis, NAc–VTA connectivity was not significantly associated with experimenter‐coded child positive mood during the parent–child interaction (β = −0.04, 95% CI [−0.24, 0.16], *p* = 0.710; see Table S4).

## 4 Discussion

In a sample of 94 infants, we examined resting‐state functional connectivity between the NAc and VTA at age one month and tested whether NAc–VTA connectivity was associated with individual differences in reward responsiveness at age six months. Our study yielded two key findings. First, we observed positive resting‐state functional connectivity between the NAc and VTA in early infancy. Second, greater NAc–VTA functional connectivity shortly after birth was associated with a lower expression of high‐intensity pleasure at age six months. Together, these findings provide preliminary evidence that early variation in reward‐related circuitry may be linked to differences in how infants express positive affect during stimulating, pleasurable experiences.

The presence of positive connectivity between NAc and VTA in early infancy suggests that core components of the mesolimbic reward system are functionally organized when assessed shortly after birth. This finding is consistent with evidence that broad functional networks and major white‐matter pathways are detectable around birth and undergo rapid postnatal refinement (De Asis‐Cruz et al. 2015; Fransson et al., 2009; Gao et al. 2017; Stephens et al. 2020). Given the rapid neural maturation occurring during this period, the observed connectivity likely represents an early and still‐developing pattern of coordinated activity that continues to reorganize throughout infancy and beyond.

The negative association between NAc–VTA connectivity and high‐intensity pleasure suggests that stronger functional coupling within this pathway may be linked to reduced behavioral exuberance in highly stimulating contexts. At six months, high‐intensity pleasure reflects infants’ observable enjoyment of stimulating activities such as bouncing, tickling, or highly interactive play, providing a behavioral index of responsiveness to salient, high‐arousal rewards. One possibility is that greater mesolimbic integration supports more efficient processing of reward cues, leading infants to require less external stimulation to experience positive affect. Alternatively, stronger connectivity could reflect early regulatory influences that dampen behavioral reactivity to high‐arousal stimuli. Although moderate levels of high‐intensity pleasure are adaptive, elevated levels have been associated with later externalizing problems, including impulsivity and attention difficulties (Brown et al. 2022; Gartstein et al. 2012, Gartstein et al. 2017). Infants with greater NAc–VTA connectivity may therefore display a more tempered form of reward responsiveness, though we did not measure later externalizing outcomes and cannot speak to whether this pattern is protective. The functional implications of early connectivity will depend on developmental context and later environmental demands.

Prior research across developmental stages has reported both positive and negative associations between mesolimbic connectivity and reward‐related behavior. For example, studies in adolescents and adults have linked stronger NAc–VTA or broader mesolimbic connectivity to greater reward sensitivity, novelty seeking, and risk‐taking (Costumero et al. 2013; Galván 2010), whereas other work has found that greater connectivity within reward circuitry is associated with lower behavioral reactivity or more efficient reward processing (Murty et al. 2018). Together, these findings suggest that the direction of associations between mesolimbic connectivity and reward behavior depends on developmental stage, likely reflecting the maturation of motivational and regulatory systems. In earlier developmental periods, when regulatory control systems are still emerging, stronger connectivity may be associated with heightened behavioral reactivity to salient rewards. In contrast, later in development, stronger connectivity may reflect more efficient coordination between reward and regulatory processes. In early infancy, when cognitive reward learning is still developing, mesolimbic circuitry is likely more closely tied to affective engagement and arousal modulation than to explicit reward seeking.

No significant association was observed between NAc–VTA connectivity and low‐intensity pleasure, which reflects enjoyment of calm or low‐arousal experiences. This specificity suggests that early mesolimbic functioning may be particularly involved in modulating responses to stimulating, high‐arousal rewards, rather than more subdued forms of pleasure. Such differentiation is consistent with evidence from older children and adults showing that distinct components of the reward system are tuned to different reward types. For example, the NAc is especially sensitive to immediate, high‐arousal rewards, whereas regions such as the orbitofrontal cortex and ventromedial prefrontal cortex contribute more to evaluating abstract, delayed, or socially contingent rewards (e.g., Bjork et al. 2004; Rademacher et al. 2014; Sescousse et al. 2013). Together, these developmental patterns suggest that variability in early mesolimbic connectivity may reflect differences in how infants regulate responses to salient stimulation, rather than simply the magnitude of reward sensitivity. These findings raise the possibility that specialization in the reward system begins in early infancy. Exploratory analyses also showed that NAc–VTA connectivity was not associated with experimenter‐coded positive mood during parent–child interaction. Although this null finding should be interpreted cautiously, it is consistent with the possibility that early mesolimbic connectivity relates more specifically to infants’ responsiveness to highly stimulating experiences than to their overall positive affective tone. Similarly, NAc–VTA connectivity was not significantly associated with negative affect, providing further preliminary evidence that the observed association with high‐intensity pleasure may not reflect broader emotional reactivity. This distinction is important because negative affect reflects a broader domain of social‐emotional temperament, including distress, fear, frustration, and sadness, rather than pleasure‐related engagement. The absence of an association with negative affect suggests that early NAc–VTA connectivity may be more closely tied to infants’ positive, approach‐oriented responses to salient stimulation than to generalized emotional reactivity.

Importantly, the high‐ and low‐intensity pleasure scales capture distinct expressions of positive affect that differ in arousal context and behavioral expression. From a developmental perspective, the present findings suggest that early mesolimbic connectivity may be particularly relevant for modulating responses to high‐arousal pleasurable experiences rather than calmer forms of positive affect. Taken together, these results highlight early variability in mesolimbic circuitry as a potential neural pathway shaping individual differences in reward sensitivity. Longitudinal research is needed to determine whether these early patterns persist across development and contribute to later risk or resilience. In particular, tracking both neural connectivity and behavioral indices of reward responsiveness across infancy and early childhood could clarify whether early NAc–VTA coupling represents a stable trait or a transient developmental feature. Understanding these trajectories will inform models of how early brain organization contributes to later motivation, affect regulation, and vulnerability to psychopathology.

### 4.1 Limitations

Several limitations should be considered. First, the racial and ethnic composition of our sample was largely representative of the region from which it was drawn, but participants had higher socioeconomic status than national averages. Whether this bounds generalization depends on whether socioeconomic context modifies the association we estimated rather than simply shifting mean levels of pleasure, which we were not able to test here (Gard et al. 2023). Second, although this study used a prospective design, with neural connectivity measured at one month and behavior at six months, each construct was assessed only once, precluding analysis of developmental change or stability across either domain. Third, although the sample size of 94 provided adequate statistical power to detect small‐to‐moderate effects, smaller associations may have gone undetected, particularly for null findings such as the association between NAc–VTA connectivity and low‐intensity pleasure.

Fourth, the internal consistency of the low‐intensity pleasure scale in the present sample was slightly below conventional thresholds, which may have attenuated observed associations and reduced statistical sensitivity to detect effects. Accordingly, the absence of a significant association should be interpreted with caution. Fifth, the primary measures of infants’ expressions of pleasure relied on caregiver report and may therefore be subject to reporting bias. Although exploratory analyses using experimenter‐coded child positive mood during parent–child interaction did not identify a significant association with NAc–VTA connectivity, this observational measure captures general affective tone rather than reward responsiveness to varying levels of stimulation. Future research should incorporate multi‐method assessments specifically designed to capture reward‐related behavior across different stimulation contexts. Finally, by focusing on NAc–VTA connectivity, we examined a theoretically central but narrow portion of the reward network; future studies may consider assessing how this circuit interacts with both cortical and limbic regions (e.g., prefrontal cortex, amygdala, and hippocampus, which also contribute to reward processing) (O'Doherty 2004; Sesack and Grace 2010).

## 5 Conclusion

In summary, we found that functional connectivity between the NAc and VTA is already present in early infancy and relates prospectively to high‐intensity pleasure. These findings point to early variation in mesolimbic circuitry as a potential marker of individual differences in reward responsiveness. Identifying such neural signatures during the first months of life may deepen understanding of affective development and inform efforts to detect atypical reward processing before behavioral symptoms emerge.

## Author Contributions

**Kathryn L. Humphreys**: writing – review and editing, supervision, funding acquisition. **Charles H. Zeanah**: writing – review and editing, supervision. **Megan M. Hare**: conceptualization, investigation, writing – original draft, methodology, writing – review and editing, funding acquisition. **Yanbin Niu**: methodology, writing – review and editing.

## Funding

This work was supported by the Klingenstein Third Generation Foundation Fellowship; Jacobs Foundation Early Career Research Fellowship (2017‐1261‐05); National Science Foundation CAREER Award (2042285); Brain and Behavior Research Foundation John and Polly Sparks Foundation Investigator Award (29593); Vanderbilt Institute for Clinical and Translational Research Grant (VR53419); Vanderbilt Strong Grant; Vanderbilt Kennedy Center Grant.

## Conflicts of Interest

The authors declare no conflicts of interest.

## Supporting information



Supporting **Table S1**. Complete‐Case Regression Results for NAc–VTA Functional Connectivity Predicting High‐ and Low‐Intensity Pleasure.
**Supporting Table S2**. Analyses Without Winsorization Predicting High‐ and Low‐Intensity Pleasure from NAc–VTA Functional Connectivity.
**Supporting Table S3**. Association Between One−Month NAc–VTA Connectivity and Negative Affect at Six Months.
**Supporting Table S4**. Association Between One‐Month NAc–VTA Connectivity and Experimenter‐Coded Child Positive Mood at Six Months.

## Data Availability

The authors have nothing to report.
