# Supplementary material for: Nucleus Accumbens and Ventral Tegmental Area Functional Connectivity as a Marker of Reward Sensitivity in Infancy
Source: Dev Sci. 2026 Sep 3;29(6):e70277. doi: 10.1111/desc.70277 (PMC13539657; doi:10.1111/desc.70277)
Supplement: Supplementary file 1 [file DESC-29-e70277-s001.docx]

**Supplemental Table S1.**

Complete-Case Regression Results for NAc–VTA Functional Connectivity Predicting High- and Low-Intensity Pleasure.

| Model | *β* | 95% CI | SE | z | *p* |  |
| --- | --- | --- | --- | --- | --- | --- |
| **Dependent Variable**: **high−intensity pleasure at six months** | | | | | | |
| NAc–VTA FC | −0.32 | −0.54, −0.10 | 0.11 | −2.82 | **.005** |  |
| fMRI Motion | 0.13 | −0.05, 0.31 | 0.09 | 1.43 | .153 |  |
| fMRI Length of Low Motion Data | −0.18 | −0.41, 0.05 | 0.12 | −1.56 | .120 |  |
| Infant Age | 0.21 | 0.02, 0.42 | 0.10 | 2.15 | **.032** |  |
| Infant Sex | 0.12 | −0.09, 0.32 | 0.10 | 1.12 | .263 |  |
| **Dependent Variable**: **low−intensity pleasure at six months** | | | | | | |
| NAc–VTA FC | −0.00 | −0.23, 0.22 | 0.11 | −0.03 | .972 |  |
| fMRI Motion | 0.07 | −0.16, 0.29 | 0.12 | 0.62 | .535 |  |
| fMRI Length of Low Motion Data | −0.01 | −0.24, 0.23 | 0.12 | −0.06 | .957 |  |
| Infant Age | −0.19 | −0.39, 0.02 | 0.10 | −1.80 | .072 |  |
| Infant Sex | −0.08 | −0.29, 0.13 | 0.11 | −0.76 | .445 |  |

***Note.*** β = fully standardized regression coefficients. SEs, z statistics, p values, and 95% CIs were obtained from the fully standardized solution. FC = functional connectivity; NAc = nucleus accumbens; VTA = ventral tegmental area. All continuous variables were z−scored before analysis. Infant age was assessed at the time of MRI scanning. Infant sex was coded as female = 0 and male = 1 and was not z-scored before model estimation; its reported coefficient is fully standardized. Analyses included only participants with complete data on all variables included in either model (N = 78).

**Supplemental Table S2.**

Analyses Without Winsorization Predicting High- and Low-Intensity Pleasure from NAc–VTA Functional Connectivity.

| Model | *β* | 95% CI | SE | z | *p* |  |
| --- | --- | --- | --- | --- | --- | --- |
| **Dependent Variable**: **high−intensity pleasure at six months** | | | | | | |
| NAc–VTA FC | −0.29 | −0.53, −0.05 | 0.12 | −2.42 | .016 |  |
| fMRI Motion | 0.11 | −0.07, 0.29 | 0.09 | 1.21 | .228 |  |
| fMRI Length of Low Motion Data | −0.15 | −0.39, 0.09 | 0.12 | −1.26 | .209 |  |
| Infant Age | 0.19 | −0.01, 0.39 | 0.10 | 1.83 | .067 |  |
| Infant Sex | 0.10 | −0.11, 0.31 | 0.11 | 0.94 | .349 |  |
| **Dependent Variable**: **low−intensity pleasure at six months** | | | | | | |
| NAc–VTA FC | −0.03 | −0.22, 0.17 | 0.10 | −0.28 | .779 |  |
| fMRI Motion | 0.07 | −0.16, 0.30 | 0.12 | 0.63 | .533 |  |
| fMRI Length of Low Motion Data | −0.01 | −0.23, 0.22 | 0.12 | −0.04 | .969 |  |
| Infant Age | −0.19 | −0.40, 0.02 | 0.11 | −1.77 | .077 |  |
| Infant Sex | −0.08 | −0.29, 0.13 | 0.11 | −0.77 | .440 |  |

***Note.*** β = fully standardized regression coefficients. SEs, z statistics, p values, and 95% CIs were obtained from the fully standardized solution. FC = functional connectivity; NAc = nucleus accumbens; VTA = ventral tegmental area. All continuous variables were z−scored before analysis. NAc–VTA functional connectivity was not winsorized in these analyses. Infant age was assessed at the time of MRI scanning. Infant sex was coded as female = 0 and male = 1 and was not z-scored before model estimation; its reported coefficient is fully standardized. Analyses used full−information maximum likelihood (N = 94).

**Supplemental Table S3.**

Association Between One−Month NAc–VTA Connectivity and Negative Affect at Six Months.

| Model | *β* | 95% CI | SE | z | *p* |  |
| --- | --- | --- | --- | --- | --- | --- |
| **Dependent Variable**: **negative affect at six months** | | | | | | |
| NAc–VTA FC | −0.23 | −0.59, 0.10 | 0.18 | −1.37 | .170 |  |
| fMRI Motion | −0.35 | −0.83, 0.10 | 0.24 | −1.54 | .124 |  |
| fMRI Length of Low Motion Data | −0.09 | −0.42, 0.24 | 0.17 | −0.54 | .593 |  |
| Infant Age | −0.03 | −0.35, 0.29 | 0.17 | −0.17 | .864 |  |
| Infant Sex | −0.10 | −0.39, 0.20 | 0.15 | −0.64 | .522 |  |

***Note.*** β = fully standardized regression coefficients. SEs, z statistics, p values, and 95% CIs were obtained from the fully standardized solution. FC = functional connectivity; NAc = nucleus accumbens; VTA = ventral tegmental area. All continuous variables were z−scored before analysis. Infant age was assessed at the time of MRI scanning. Infant sex was coded as female = 0 and male = 1 and was not z-scored before model estimation; its reported coefficient is fully standardized. Analyses used full−information maximum likelihood (N = 94).

**Supplemental Table S4.**

Association Between One-Month NAc–VTA Connectivity and Experimenter-Coded Child Positive Mood at Six Months.

| Model | *β* | 95% CI | SE | z | *p* |  |
| --- | --- | --- | --- | --- | --- | --- |
| **Dependent Variable**: **experimenter**-**coded child positive mood at six months** | | | | | | |
| NAc–VTA FC | −0.04 | −0.24, 0.16 | 0.10 | −0.37 | .710 |  |
| fMRI Motion | 0.15 | −0.07, 0.35 | 0.11 | 1.35 | .177 |  |
| fMRI Length of Low Motion Data | −0.13 | −0.28, 0.02 | 0.07 | −1.68 | .093 |  |
| Infant Age | −0.06 | −0.21, 0.09 | 0.07 | −0.83 | .407 |  |
| Infant Sex | 0.08 | −0.16, 0.32 | 0.12 | 0.65 | .517 |  |

***Note.*** β = fully standardized regression coefficients. SEs, z statistics, p values, and 95% CIs were obtained from the fully standardized solution. FC = functional connectivity; NAc = nucleus accumbens; VTA = ventral tegmental area. All continuous variables were z−scored before analysis. Infant age was assessed at the time of MRI scanning. Infant sex was coded as female = 0 and male = 1 and was not z-scored before model estimation; its reported coefficient is fully standardized. Analyses used full−information maximum likelihood (N = 94).
